# Shared mechanisms of organ fibrosis

**DOI:** 10.1172/jci.insight.200952

**Published:** 2026-07-08

**Authors:** Benjamin D. Humphreys

**Affiliations:** 1 Division of Nephrology, Department of Medicine, and; 2 Department of Developmental Biology, Washington University in St. Louis, St. Louis, Missouri, USA.

## Abstract

Organ fibrosis involves a complex interplay between diverse cell types and signaling pathways that ultimately leads to the pathologic accumulation of excessive extracellular matrix, subsequently resulting in organ dysfunction. In recent years, the first drugs for the treatment of idiopathic pulmonary fibrosis have been approved; however, there is a major unmet need for effective antifibrotic therapies across organs. Despite the complexity of the fibrotic process in different tissues, certain features are shared and may form the basis for future therapeutic strategies. This Review will highlight these shared characteristics, cell states, and signaling pathways across organs with the goal of highlighting potential antifibrotic strategies.

## Introduction

Fibrosis is a process of pathological remodeling triggered by repetitive or severe injury, resulting in the accumulation of extracellular matrix (ECM), the loss of parenchyma, and ultimately organ dysfunction and failure. Fibrotic diseases are responsible for a substantial fraction of deaths in the industrialized world, and yet, there are only two approved therapies that directly target fibrosis, highlighting an enormous unmet medical need. Knowledge concerning the cellular and molecular mechanisms driving fibrosis has increased considerably over the last decade, with the prospect of novel therapies that target common mechanisms of fibrosis across organs now on the horizon (1). This Review will focus on recent findings over the last five years identifying common cell types, cell states, and core pathways that are shared across organs with the goal of highlighting potential antifibrotic therapeutic strategies. Emphasis will be placed on heart, liver, lung, and kidney. While inflammation plays a critical shared role in organ fibrosis, it is reviewed elsewhere in this issue of *JCI Insight* and will not be a primary focus here.

## Triggers of fibrosis

Fibrosis results from the dysregulated activation of tissue repair programs. Triggers are disparate, including genetic causes, environmental exposures, toxic or ischemic insults, and chronic inflammation, and injury is typically repetitive or sustained, causing parenchymal damage, cell death, and subsequent inflammation (2–4). Regardless of cause, an important shared feature of fibrotic triggers across organs is the injury-induced emergence of pathologic parenchymal cell states. These represent surviving cells that adopt pro-inflammatory phenotypes driving local inflammation and ultimately fibrosis. Unlike homeostatic repair, in which injured parenchyma activates productive responses that restore function and resolve inflammation, pathological fibrosis is characterized by persistence of these maladaptive cell states. In lung, kidney, liver, and heart, these correspond to alveolar epithelial, tubular epithelial, hepatocyte, and cardiomyocyte cell states, respectively. Although the marker genes distinguishing these pathological cell states differ between organs, shared features include loss of terminal differentiation markers and gain of pro-inflammatory and mesenchymal gene programs.

Support for the primacy of parenchyma in initiating the fibrotic process derives from both genetic defects in intrinsic proteins and GWAS, where many fibrosis risk alleles are located near genes expressed in parenchymal cells (5). For example, variants associated with idiopathic pulmonary fibrosis (IPF) expressed in airway epithelial cells include *MUC5B*, which encodes mucin, *DSP* encoding desmoplakin, and surfactant genes *SFTPB*, *SFTPC*, and *SFTPA1/2* (6). Recent genetic evidence also links IPF susceptibility to the airway epithelium (7, 8). Coding variants in over 100 genes are linked with the various cardiomyopathies, and the majority are sarcomere or cytoskeletal proteins (9). Autosomal recessive forms of cirrhosis are caused by defects in hepatocyte proteins, for example α1-antitrypsin disease, Wilson’s disease, inborn errors of metabolism, and hereditary hemochromatosis (10, 11). Variants in MUC1 and UMOD, expressed in thick ascending limb, cause chronic tubulointerstitial disease. Similarly, GWAS variants associated with estimated glomerular filtration rate (eGFR) localize primarily to tubular epithelia, with the majority linked to proximal tubule (PT) genes (12, 13).

## Epithelial and parenchymal injury

In recent years, single-cell analyses have characterized injured cell states at molecular resolution. In IPF, hyperplastic alveolar cells colocalize in fibrogenic foci; coexpress basal epithelial, mesenchymal, senescence, and developmental markers; and have more recently been recharacterized as “aberrant basaloid cells,” reflecting their origin as basal cells that have migrated into the alveolar space (14–16). Aberrant basaloid cells lose expression of the basal cell marker keratin 5 (KRT5), upregulate expression of KRT17, and are profibrotic with enhanced capacity to form bronchospheres (17). These aberrant cells colocalize with activated fibroblasts and myofibroblasts and express integrin α_V_β_6_, a specific activator of latent TGF-β, and thus are a potential therapeutic target in IPF (18, 19). An important paracrine mediator is IL-1α, which is released from damaged alveolar epithelial cells and activates pro-inflammatory and profibrotic pathways in adjacent fibroblasts (20).

During the repair process after acute kidney injury (AKI), a small proportion of injured PT cells fail to undergo full repair without restoration of a healthy PT phenotype (21–24). These so-called failed repair PT (FR-PT; also called “maladaptive PT” in the literature) cells are characterized by expression of Vcam1 and take on a pro-inflammatory, profibrotic, senescence-associated secretory pathway (SASP) phenotype, thus providing a possible mechanism for AKI to chronic kidney disease (CKD) transition (24–26). FR-PT cells exhibit a very strong NF-κB transcriptional signature (27, 28), suggesting that this pro-inflammatory transcription factor may be driving the inflammatory phenotype. Originally described due to their presence after AKI, FR-PT cells are also present in apparently healthy kidneys and increase with aging, in diabetic nephropathy, in autosomal dominant polycystic kidney disease, and in human kidney transplants (29–31). These findings suggest that failed repair cells may accumulate as the kidney undergoes chronic subclinical injury during homeostasis. Thus, FR-PT cells are a possible convergence point through which acute or chronic insult can lead to progressive damage and fibrosis in susceptible individuals. Recent work has shown that FR-PT cells accumulate the highest frequency of somatic mutations in the kidney, raising intriguing parallels with clonal hematopoiesis of indeterminate potential in the hematopoietic system (32, 33).

Huang et al. recently extended this concept by showing that AKI triggers a burst of somatic mtDNA mutations that persist into the chronic phase and sensitize the kidney to further injury and fibrosis (34). In a UK Biobank cohort of nearly 370,000 individuals, mtDNA burden was independently associated with both CKD severity and future AKI risk. These findings are consistent with the feed-forward model in which FR-PT cells accumulate somatic mutations that perpetuate vulnerability to future kidney damage and fibrosis.

Transitional and failed repair epithelial cells are not passive by-products of incomplete regeneration; they actively promote fibrosis through their secretome. Kidney FR-PT cells upregulate TGF-β, CTGF, and CCL2, directly recruiting fibroblasts. A comparable paracrine program operates in IPF, where aberrant basaloid cells secrete profibrotic myofibroblast cytokines acting in adjacent fibrogenic foci. Both cell populations are characterized by a SASP phenotype, which amplifies fibroblast activation and proliferation. These findings suggest that the epithelial cell/fibroblast signaling axis may be a conserved therapeutic target across organs.

Single-cell studies of heart failure have identified *NPPA*, encoding natriuretic peptide A, and *NPPB*, encoding brain natriuretic peptide, as significantly upregulated in diseased cardiomyocytes during heart failure (35, 36). Like parenchyma in other organs, the cardiomyocyte stress response also includes upregulation of dedifferentiation markers (37). A large multiomic analysis provided further markers of the cardiomyocyte transcriptional stress signature (*NPPB*, *LMCD1*, *XIRP2*, *ACTA1*, and *PFKP*) (38). Ligand receptor analysis of the stressed cardiomyocyte niche suggested that immune and vascular cells secreted TGF-β superfamily members that were received by activated fibroblasts and stressed cardiomyocytes. In fact, stressed myocytes upregulated genes encoding receptors for IL-6, TNFSF12, and IL-1, which are all associated with the pathogenesis of heart failure (39). Larger single-cell atlases are able to distinguish multiple cardiomyocyte stress states, corresponding either to ventricular location or to cell function, for example apoptosis score (40).

Depending on the etiology, liver fibrosis originates in either the pericentral or periportal region, reflecting the site of primary hepatocyte injury. Damage to hepatocytes causes cell death and inflammation, with release of soluble mediators acting as damage-associated molecular patterns (DAMPs) that activate surrounding parenchymal and nonparenchymal cell types (41). For example, acetaminophen overdose induces centrilobular necrosis and hepatocyte injury, which Umbaugh et al. characterized at the single-cell level. They described hepatocyte cell death closest to the central vein, with surviving hepatocytes in adjacent regions displaying adaptive (oxidative stress) responses and maladaptive (MAPK, glutamine synthetase) responses (42). Wang et al. have shown through lineage tracing that surviving pericentral hepatocytes mediate liver regeneration via upregulation of mTOR/4E-BP1 axis and lactate dehydrogenase A: whereas monocyte recruitment and secretion of TGF-β1 in damaged areas suppresses hepatocyte proliferation and promotes fibrosis (43). A separate study identified p21^+^ hepatocytes after acetaminophen injury that directly surround necrotic areas and secrete CXCL14, inhibiting regeneration and promoting fibrosis (44). As in other organs, the responses on nonparenchymal cell types to liver necrosis and injury are critical mediators of fibrosis, for example scar-associated and profibrogenic TREM2^+^CD9^+^ macrophages; ACKR^+^PLVAP^+^ activated endothelial cells, which recruit leukocytes; and the activation, proliferation, and differentiation of hepatic stellate cells (HSCs; described in detail below) (45).

These single-cell analyses across lung, kidney, heart, and liver reveal a convergence: despite diverse upstream insults, parenchymal cell populations in the injury-repair microenvironment share a common profibrotic trajectory, which is now definable at molecular resolution. Targeting these conserved parenchymal cell states represents a promising therapeutic strategy across organs.

## The fibrotic neighborhood in organ fibrosis

A common paradigm in organ fibrosis is that resident mesenchymal cells, fibroblasts, and pericytes differentiate upon tissue injury into contractile myofibroblasts that secrete matrix proteins and impair organ function. This process of myofibroblast differentiation occurs in a fibrotic microenvironment with shared spatial characteristics across organs. These fibrotic niches are characterized by injured parenchyma, activated myofibroblasts, and circulating monocytes and macrophages — all secreting and responding to signals from neighboring cells and the microenvironment to drive fibrosis. Rather than progress in a uniform, even manner across a tissue, these fibrotic niches are typically patchy and discontinuous with focal areas of high ECM gene expression. Recent work incorporating spatial technologies has greatly expanded our understanding of spatial heterogeneity of fibrotic neighborhoods in organ fibrosis (46).

In a multiomic and spatial analysis of human CKD, Abedini et al. described a fibrotic microenvironment gene signature, present in a patchy distribution in the kidney cortex, and showed that it could be used on an independent cohort of kidney samples to predict the degree of fibrosis and lower eGFR (47). Samples with the highest fibrotic gene score had a much higher chance of developing progressive CKD or end-stage renal disease, implying a causal role for the fibrotic microenvironment in driving CKD progression. After severe AKI, patients are at risk of developing CKD, the so-called AKI to CKD transition. Dixon et al. leveraged the Visium spatial transcriptomics platform at late time points after murine AKI and found spatial enrichment of T cells and macrophages adjacent to persistently injured tubules (48). Three higher resolution spatial studies (employing CARTANA, SeqFISH, or Visium HD) of the AKI to CKD transition found these immune cells adjacent to Vcam1^+^ PTs characterized by dedifferentiation and upregulation of pro-inflammatory and profibrotic genes (49–51). These observations have been supported by human omics and spatial studies that identified similar fibrotic microenvironments characterized by maladaptive PT or thick ascending limb and activated macrophages, T cells, and myofibroblasts (25).

Spatial heterogeneity is also a hallmark of IPF histopathology, wherein a densely fibrotic region may be adjacent to areas of preserved alveolar architecture. Another cardinal feature of IPF lungs are small aggregates of proliferating fibroblasts that secrete matrix proteins with an overlying layer of hyperplastic alveolar epithelia collectively termed fibrogenic foci. These represent a transition zone between normal lung and abnormal fibrotic lung and are hypothesized to represent the so-called leading edge of fibrosis.

A recent comprehensive spatial transcriptomic study examined these IPF microenvironments in detail and suggests that the earliest events in IPF are not an influx of inflammatory cells or activation of fibroblasts, but rather disruption of the alveolar epithelium and adjacent capillary network before other structural remodeling can be detected (52). Regions were identified where KRT5^–^KRT17^+^ aberrant basaloid cells apparently lifted off the basement membrane in areas adjacent to activated fibroblasts. Similar desquamated epithelia in distal bronchioles have been reported in fresh IPF biopsies (53). The significance of this finding requires further investigation, but the authors speculate that the resulting exposed basement membrane may permit migration of fibroblasts into the airway, leading to airway obstruction and/or cystic structures distally. Finally, Vannan et al. showed that in morphologically preserved nonfibrotic areas of IPF lungs, a molecular signature associated with normal alveoli was virtually absent, indicating that substantial injury occurs at the molecular level well before histologic remodeling can be detected (52), similar to prior findings (54).

Multiomic analyses have defined similar distinct fibrotic microenvironments or zones during cardiac remodeling after myocardial infarction (MI). Kuppe and colleagues analyzed human samples and defined a fibrotic zone enriched for fibroblasts and SPP1^+^ macrophages, consistent with known signaling relationships between these two cell types in fibrosis (55–57). While this fibrotic zone was defined by unsupervised multiomic analysis, it corresponded spatially to regions with histologic fibrosis. Amrute et al. have further dissected macrophage-fibroblast crosstalk in cardiac fibrosis, identifying a distinct fibroblast lineage characterized by expression of fibroblast activation protein (FAP) and periostin (POSTN) that does not contribute to the ACTA2^+^ myofibroblast lineage. Ligand-receptor analyses suggested IL-1β production by CCR2-expressing macrophages drives Fap/Postn-positive fibroblast activation in fibro-inflammatory foci. Strong experimental evidence supported this model, as deletion of the IL-1β receptor in Fap/Postn fibroblasts, *IL1B* deletion in CCR2^+^ macrophages, or neutralizing IL-1β Ab all prevented cardiac fibrosis in experimental models. Moreover, *IL1B* deletion in CCR2^+^ macrophages improved cardiac function (58).

As alluded to briefly above, the liver is composed of anatomical structures called lobules, within which hepatocytes are organized into three zones: periportal hepatocytes or zone 1, midlobular hepatocytes or zone 2, and pericentral or zone 3 (59). Periportal hepatocytes localize closest to the portal vein and are exposed to a more oxygen- and nutrient-rich environment. Pericentral hepatocytes have higher rates of glycolysis and lipogenesis, reflecting the depletion of oxygen and nutrients in their environment. Recent spatial analyses have examined gene expression and cell type changes within the lobule during liver fibrosis. Watson et al. designed a 317-gene panel and employed the high-resolution spatial transcriptomic modality multiplexed error robust fluorescent in situ hybridization (MERFISH) to interrogate healthy and fibrotic human liver (60). In healthy tissue, three hepatocyte clusters were transcriptionally distinguished and mapped to the lobule, corresponding to zones 1 through 3. Hepatocytes can become multinucleated, and one third of the healthy hepatocytes were multinucleated; however, the distribution of these hepatocytes did not differ across zones (59).

In fibrotic liver samples, hepatocyte zonation was largely preserved, whereas hepatic stellate cells were located diffusely throughout the lobule and activated macrophages had a periportal distribution (60). The latter observation bears similarity to a separate Visium-based study in human primary sclerosing cholangitis, where the T cells, B cells, NK cells, and activated macrophages were shown to localize to the periportal region (61). Two independent studies localized a cholangiocyte-like hepatocyte cell state that emerges at the scar-parenchyma interface. Using Visium and single-cell multiomics, Hammond and colleagues identified EPCAM/SOX9^+^ hepatocytes at the scar interface and implicated the transcriptional regulator ONECUT1 as a possible regulator of this fibrosis-associated cell state (62). Andrews et al. found a population of fibrosis-associated hepatocytes that had downregulated zonal markers and upregulated inflammatory and cholangiocyte genes, potentially representing a similar hepatocyte cell state (61). Transdifferentiation of hepatocytes into cholangiocytes has previously been shown to be TGF-β dependent (63, 64), and TGF-β expression could be detected at the scar-hepatocyte border.

## Fibroblast heterogeneity across organ fibrosis

The widespread application of single-cell technologies in recent years has provided strong support for the long-held hypothesis that organ-specific fibroblasts possess different functional capacities (65). Fibroblasts localize within connective tissue and synthesize ECM proteins, such as collagens, elastin, and fibronectin. These cells interact with epithelial, immune, and vascular cells to maintain normal tissue homeostasis and can react to tissue injury by adopting a contractile phenotype — the myofibroblast (66). In disease, myofibroblast states are the key effectors driving fibrosis (67–71). Fibroblast classification is complicated, as stromal subtypes are typically defined by combinations of markers rather than expression of a single unique gene. This complication is shared between organs as well, although some evidence implicates murine *Pi16^+^* and *Col15a1^+^* fibroblast subtypes as universal in healthy tissue (72). Another exception is expression of the hedgehog (Hh) pathway member glioma-associated oncogene 1 (Gli1), which marks a pericyte and perivascular fibroblast population that drives fibrosis in kidney, heart, lung, liver, and bone marrow (73–75). These Gli1^+^ progenitors differentiate into myofibroblasts, and their genetic ablation ameliorates fibrosis across organs. The advent of single-cell technologies has greatly aided the traditional morphological and spatial classifications of fibroblast subtypes (76).

Largely due to recent results from single-cell investigations, it has become clear that there is an underappreciated and profound degree of fibroblast heterogeneity between organs and during pathologic fibrosis (76). This is an important observation, because evidence suggests that specific fibroblast subsets may be more responsible for ECM secretion than others, and consequently, different subsets may represent more specific therapeutic targets. Because fibroblasts are typically embedded in matrix, they can be underrepresented in single-cell studies due to inadequate enzymatic dissociation. As studies have gotten larger, more distinct subclusters of stromal cells have been revealed (25, 38, 77–79).

Some investigators have used flow cytometry purification strategies to enrich for stromal cells prior to single-cell transcriptional analysis (80). One study leveraged *Col1a1*-EGFP mice to harvest all collagen-producing cells during murine pulmonary fibrosis. Twelve distinct subclusters were identified and broadly annotated as alveolar fibroblasts, adventitial fibroblasts, peribronchial fibroblasts, smooth muscle cells, and pericytes (81). The highest collagen-producing subcluster was marked by expression of collagen triple helix repeat containing 1 (CTHRC1), and these cells had high migration capacity, and following adoptive transfer into injured lung, had high engraftment potential at the injury site, suggesting that they may drive pulmonary fibrosis. CTHRC1^+^ pathological fibroblasts were also identified in a human lung single-cell atlas in fatal COVID-19 infection, along with four other fibroblast subtypes (82). Recent genetic lineage analyses conclusively implicate alveolar fibroblasts as the primary source of pro-inflammatory and profibrotic activated fibroblasts after injury (83). Moreover, alveolar cell ablation alone reduced alveolar stem cell (AT2 cell) numbers and exaggerated responses to acute lung injury, highlighting the role of these cells in maintaining alveolar homeostasis.

The major mesenchymal cell type in liver is the HSC, a resident pericyte that stores vitamin A and resides in the perisinusoidal space between hepatocytes and endothelium (68). HSCs are the primary cell type that gives rise to myofibroblasts during fibrosis (84, 85). Similar to AT2 cells in the lung, HSCs also regulate homeostasis, for example by controlling hepatocyte zonation through secretion of the Wnt amplifier R-spondin 3 (86). HSCs also express hepatocyte growth factor, which acts on hepatocytes to protect them from toxic or high-fat diet–induced liver injury (87, 88).

It is increasingly recognized that HSC subsets exist both during liver homeostasis and in fibrosis. In health, HSC heterogeneity largely tracks spatially with lobule zonation. For example, an nerve growth factor receptor-high (NGFR^hi^) HSC subpopulation is enriched in the periportal region, whereas an a disintegrin and metalloproteinase with thrombospondin motifs 12^hi^ (ADAMTS12^hi^) subpopulation is pericentral (89). This study further implicated central vein–associated HSCs as the predominant HSC subtype contributing to the pathogenic collagen-producing myofibroblast pool during acute CCl_4_-induced liver injury. By contrast, in biliary liver diseases, such as primary sclerosing cholangitis, fibrosis initiates in the portal area (90). In this scenario, the NGFR^hi^ periportal HSC subpopulation may be the primary contributor to the myofibroblast pool, although this requires experimental confirmation.

Five fibroblast subpopulations have been identified in healthy mouse heart, including one characterized as Pdgfra^+^Ly6a/Sca1^hi^ that resides in a hypoxic niche and may preferentially proliferate in response to injury (91). A similar Sca1^+^ fibroblast subtype expressed transcription factor Hic1, and *Hic1* deletion in the stromal lineage led to spontaneous fibrosis, suggesting that Hic1 normally functions to maintain fibroblast quiescence (92). A recent study used a mouse model of MI and isolated PDGFR-α and DDR2 double-positive fibroblasts from infarct and remote zones. From the 69,705 single cells, a total of 13 fibroblast populations were identified in health and after MI (93). One subpopulation specifically expressed CD248 and expanded at late time points. These CD248^+^ fibroblasts recruit profibrotic CD4^+^ T cells, and genetic deletion or inhibition of CD248 with a mAb prevented cardiac fibrosis. Intriguingly, this fibroblast subtype is also enriched in lung and kidney fibrosis (93). Seven distinct fibroblast subtypes could be distinguished in human heart, with three of these posited to respond to stress (94).

Like other organs, stromal cells play important roles in maintaining kidney homeostasis. Ablation of kidney pericytes is sufficient to trigger capillary rarefaction and tubule injury, for example (95). During fibrosis, kidney injury triggers pericytes and fibroblasts to proliferate and differentiate into heterogeneous myofibroblast subtypes with distinct regional localization and functional profiles (96). A large multiomic analysis identified cortical versus medullary fibroblasts and four distinct myofibroblast populations with unique roles in migration, inflammation, metabolism, and ECM deposition (97). NKD2 is selectively expressed in kidney myofibroblasts with high ECM expression but not in other stromal or epithelial cell types. Functional experiments have suggested that NKD2 promotes fibrosis, highlighting it as a therapeutic target (98). A recent study has shown that ADAMTS12 is strongly upregulated in kidney pericytes after injury. This metalloprotease cleaves the large ECM protein hemicentin-1, allowing activated pericytes to migrate out of the perivascular space, differentiate into myofibroblasts, and promote fibrosis (99).

A key unresolved question regarding fibroblast heterogeneity is the degree to which molecularly distinct fibroblast subpopulations have unique functional characteristics. Alternatively, do these different groups represent a transient state on a developmental trajectory or a reactive injury response? Simply put, just because a fibroblast subpopulation forms a subcluster in a single-cell RNA-seq experiment does not guarantee that it is functionally distinct from a neighboring subcluster. A related, unresolved question is the degree to which fibroblast subtypes may be specified by external cues from their spatial niche versus cell-autonomous factors. For example, if one fibroblast subtype is ablated, can a different subtype migrate into the empty niche and differentiate into the lost cell, guided by cues from the microenvironment? To what degree are these cell subtypes plastic? These questions await further investigation with more traditional and emerging genetic lineage tracing methodologies (100).

## Shared core fibrotic signaling pathways

**Figure 1:**
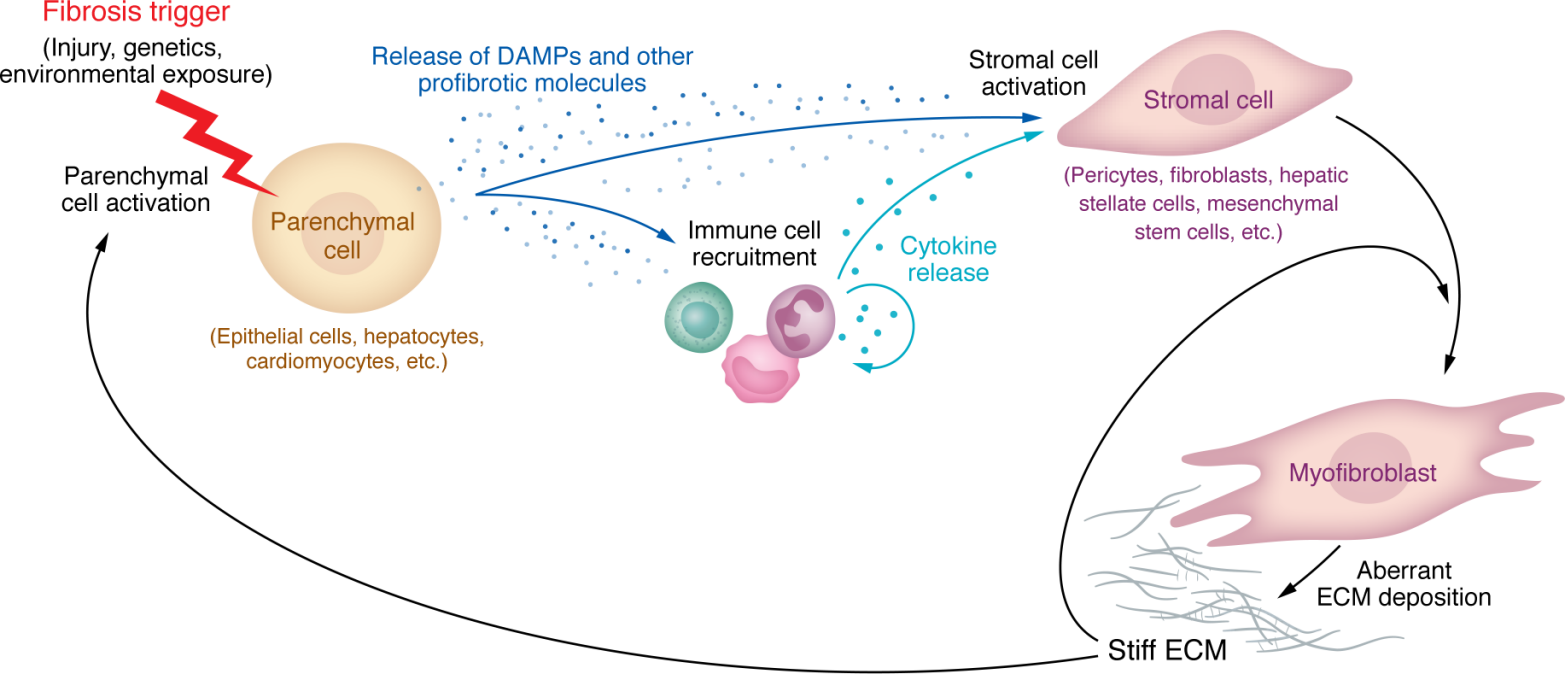
Shared pathways of fibrosis development. In all organs, fibrosis can be triggered by a variety of insults, including injury, environmental exposures, and genetic predisposition. In response to the inciting trigger, parenchymal cells are activated and/or damaged, resulting in the release of profibrotic factors. These parenchymally derived factors in turn promote recruitment of immune cells and activation of stromal cell populations. Activated stromal cells then convert or support conversion to pathogenic myofibroblasts. Myofibroblasts secrete high levels of ECM that results in tissue stiffness, which in turn further promotes parenchymal and stromal cell activation to further exacerbate fibrosis.

Myriad tissue- and cell-specific molecular mechanisms drive fibrosis, many of which are summarized elsewhere (for a review, see refs. 101–103). Despite diverse signaling pathways driving fibrosis across organs, there are also a variety of shared mechanisms (Figure 1) (103). Tissue stiffness and mechanosensing in fibrosis are explored in detail below, along with a brief review of other shared signaling pathways that is not fully comprehensive. The central role of inflammation, which is common across all types of fibrosis, is not emphasized here, as it is covered elsewhere in this issue. Similarly, TGFB is a master regulator of organ fibrosis, has been reviewed extensively (104–106), and is not covered in depth here.

### Mechanosensing and fibrosis.

Tissue stiffness is a shared biomechanical signature of all chronic fibrotic diseases and has been historically viewed as a consequence of rather than a cause of fibrosis. However, a substantial body of work implicates physical force as an amplifier of fibrosis across organs. Tissue injury causes changes in cell-cell and cell matrix–dependent mechanoregulation of barrier function, parenchymal cell activation in response to stretch or shear, and fibroblast activation in response to ECM stiffness (107, 108). Cells sense changes in mechanical forces primarily (but not exclusively) through integrin clustering after binding to ECM, which links to the cell cytoskeleton, subsequently creating stress fibers and recruiting intracellular adaptor proteins to form focal adhesions. Injury itself upregulates expression of certain integrins, such as the epithelium-restricted integrin α_v_β_6_, which is induced in pulmonary, liver, and kidney fibrosis (109, 110). Integrins activate downstream profibrotic pathways, several of which are shared across organs. Most notably, integrins can activate latent TGFB1 and TGFB3 by binding to the RGD motif on the latency-associated peptide (LAP), which anchors these two TGFB isoforms to ECM. Cell contraction transmits force through integrins to LAP, distorting its conformation and releasing TGFB (111). As a recent example of this phenomenon, Wu et al. deleted the RhoGTPase and actin polymerization regulator *Cdc42* in AT2 cells. In the absence of CDC42, AT2 cells could not generate AT1 cells, and after injury, the AT2 cells were exposed to elevated mechanical tension, activating a TGFB signaling loop and driving progressive pulmonary fibrosis (112).

Tissue stiffness also potently activates fibroblasts to differentiate into myofibroblasts, and this is true across organs. Fibroblasts express the α_v_ integrin subunit in combination with various β integrin subunits. Deletion of α_v_ in fibroblasts using *Pdgfrb*-Cre inhibited liver, lung, and pulmonary fibrosis, revealing a conserved α_v_ integrin–dependent profibrotic pathway (113). Recently Cho and colleagues demonstrated that activated cardiac fibroblasts sense microenvironment stiffness through the focal adhesion-associated kinase SRC (Figure 1). Importantly, they could inhibit fibroblast matrix mechanosensing by pharmacological inhibition of SRC, which, coupled with TGFB inhibition, induced activated fibroblast regression and ameliorated contractile dysfunction in a mouse heart failure model (114). This important finding established a new mechanotherapeutic approach that could be relevant in other organs. Whether similar SRC-dependent mechanosensing pathways exist in organs besides the heart requires investigation. Tissue-resident macrophages also possess mechanosensing properties, which are intriguingly integrin independent. Meizlish and colleagues report that macrophages integrate microenvironment stiffness by intracellular cytoskeletal dynamics, suppressing a prorepair gene expression program and exacerbating pulmonary fibrosis (115). Clearly this is another mechanosensing pathway with potential relevance across organs.

**Figure 2:**
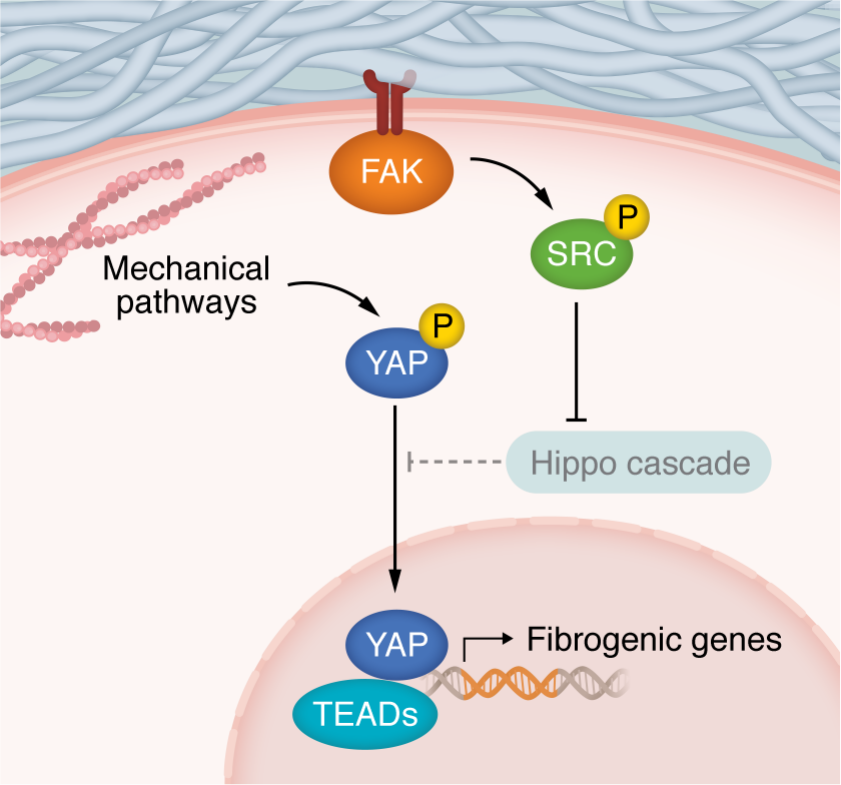
Mechanosensation and fibrosis. Cells detect stiffness primarily through integrin receptors that bind ECM. This activates FAK, which phosphorylates SRC, relieving inhibition of the Hippo pathway. This causes YAP dephosphorylation, translation into the nucleus, and transcription of profibrogenic genes. Stiffness within the cell is also detected by mechanical pathways, which can independently activate YAP. TEAD, TEA domain transcription factor.

Finally, the YAP/TAZ pathway integrates mechanical cues to regulate cell behavior, and evidence supports important roles for this pathway in fibrosis across organs. YAP/TAZ are transcription factors that are primarily activated by integrins/focal adhesion kinase (FAK) and by cytoskeletal tension, but they can also be activated by Wnt, Hippo, and GPCRs (Figure 2) (116). Activated YAP/TAZ translocates into the nucleus and recruits TEAD family members to drive transcription of genes that regulate cell proliferation, cell protection, and metabolism, among others. YAP/TAZ are activated in myofibroblasts early after injury across organs (117–119), and genetic deletion of both genes in myofibroblasts prevented kidney, lung, and liver fibrosis, whereas activation of YAP/TAZ — by deleting the inhibitory Hippo pathway kinases LATS1 and LATS2 in myofibroblasts — exacerbated kidney and lung fibrosis (120). A separate study showed that YAP and TAZ deletion in cardiac fibroblasts reduces MI-associated fibrosis (121). Pharmacological targeting of YAP/TAZ or TEAD for the treatment of organ fibrosis is a promising approach, but it remains in preclinical development (122). For example, Wagner et al. recently identified YAP-dependent expression of ECM cross-linker lysyl oxidase in AT2 cells and showed that pharmacologic inhibition of YAP with verteporfin attenuated bleomycin-induced pulmonary fibrosis and improved survival in a mouse model. Moreover, verteporfin also reduced fibrosis in a human lung explant model (123).

A very recent study examined how dysregulated hepatic cholesterol metabolism drives fibrogenesis in metabolic dysfunction–associated steatohepatitis (MASH). MASH livers were characterized by decreased expression of EH-domain-binding protein 1 (EHBP1), a protein involved in intracellular sorting (124). EHBP1 normally promotes sortilin-mediated PCSK9 secretion, which increases LDL receptor degradation, reduces hepatocyte LDL uptake, and decreases profibrogenic TAZ. Further the TNFA/PPARA pathway was shown to suppress EHBP1 in MASH, which subsequently promotes fibrogenesis through increased TAZ expression, thereby providing a link between inflammation, EHBP1-mediated cholesterol metabolism, and fibrogenesis (124).

### Other shared signaling pathways: Hh and Wnt.

The Hh pathway is evolutionarily conserved and plays key roles in tissue patterning during embryonic development (125). In adult homeostasis, the Hh pathway is quiescent but becomes activated after kidney, liver, and lung injury and promotes fibrosis. In the kidney, injury and inflammation induce PT expression of Indian hedgehog, which acts on Gli1^+^ interstitial fibroblasts and myofibroblasts to drive activation, proliferation, and fibrosis (73, 74, 126). A similar scenario occurs in diverse forms of liver fibrosis, where injured hepatocytes upregulate expression of Hh ligands, which act primarily on HSCs, driving activation into fibrogenic myofibroblasts (127, 128). In pulmonary fibrosis, epithelial injury causes induction of Sonic hedgehog, which both activates myofibroblast differentiation and inhibits myofibroblast apoptosis (129). A phase IIa trial of the oral Hh inhibitor taladegib for the treatment of IPF was recently published and showed an acceptable safety profile with clinical improvement, thus supporting advancement to a phase IIb trial (130).

Similar to the Hh pathway, the Wnt/β-catenin pathway is also required for development, largely dispensable during homeostasis, but reactivated in regeneration and fibrosis (131). In IPF, Wnt/β-catenin signaling is upregulated and, in epithelia, drives AT2 to AT1 transdifferentiation as well as fibroblast activation, differentiation, and proliferation (132). In the heart, deletion of β-catenin in fibroblasts abrogates cardiac fibrosis and improves cardiac function (133). Wnt/β-catenin signaling is activated during liver fibrosis, but its effects are context dependent, with evidence supporting both exacerbation of and amelioration of fibrosis depending on the condition (134). Wnt/β-catenin signaling increases after kidney injury, and overexpression of Wnt ligands is sufficient to promote fibrosis (135), whereas inhibition of Wnt ligand secretion protects against fibrosis (136). By contrast, tubule-specific stabilization of β-catenin protected against the development of kidney fibrosis, suggesting cell- and context-specific roles for this pathway in kidney injury responses (137).

## New approach methodologies for fibrosis research

Progress in fibrosis research is also being enabled by new approach methodologies (NAMs) that provide human-relevant, tractable experimental platforms that are intermediate between bulk tissue analyses and in vivo models (138, 139). Thin ex vivo tissue slices that preserve multicellular architecture permit functional and omics interrogation while retaining cell type compositions of the intact organ (140–142). Human organoid systems are widely available and enable mechanistic dissection of fibrotic transitions, though the degree to which the organoids achieve adult cellular maturity varies considerably (16, 143–146). Organ-on-chip platforms add dynamic flow and mechanical cues that are challenging to replicate in conventional culture (147). Together, NAMs bridge the gap between static single-cell atlases and in vivo models, providing human-specific contexts in which candidate therapeutic targets or novel mechanisms can be validated before clinical translation. Importantly, these NAMs are explicitly supported by the FDA Modernization Act 2.0 (148).

## A future therapeutic direction for treating fibrosis across organs

**Figure 3:**
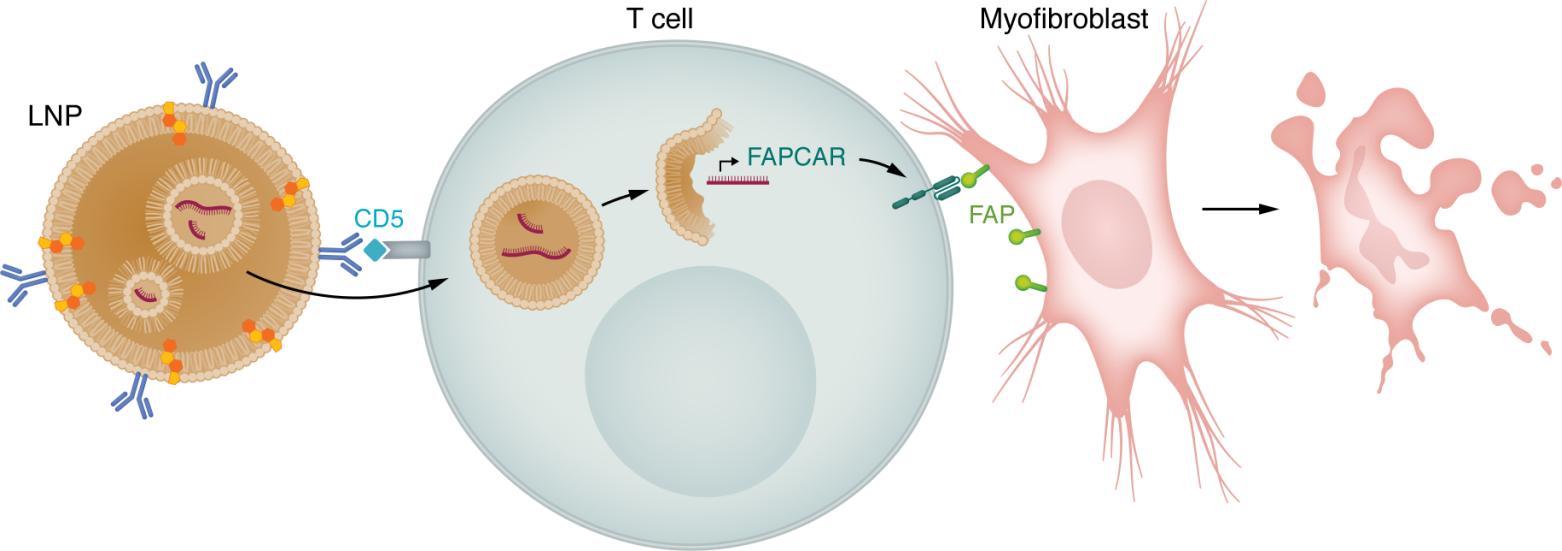
Generation of antifibrotic CAR T cells in vivo. A lipid nanoparticle (LNP) containing mRNA is targeted to T cells using anti-CD5. The LNP undergoes endocytosis, and LNP degradation releases mRNA encoding the fibroblast activation protein–targeted chimeric antigen receptor T cells (FAPCAR), which is translated and inserts into the plasma membrane. Binding to FAP expressed on activated myofibroblasts causes myofibroblast killing and reversal of fibrosis.

One of the most exciting new therapeutic avenues in treating fibrosis is the potential of chimeric antigen receptor (CAR) T cells, originally developed to treat cancer, to kill nonmalignant myofibroblasts. CAR T cells are designed to recognize and bind specific membrane receptors on target myofibroblasts, resulting in cell killing. Early studies generated CAR T cells through ex vivo transduction with a retroviral vector containing DNA that instructed T cells to bind FAP, which is specifically expressed on activated myofibroblasts during cardiac fibrosis (1). Adoptive transfer of FAP-expressing CAR T cells both reduced cardiac fibrosis and improved cardiac function, providing the first proof of principle for immunotherapy of organ fibrosis. A subsequent study generated CAR T cells targeting urokinase-type plasminogen activator receptor (uPAR), which is upregulated on senescent cells, including HSCs during liver fibrosis. Adoptive transfer of uPAR-specific CAR T cells successfully eliminated pro-inflammatory myofibroblasts in both CCl_4_ and MASH liver fibrosis models (149). This approach has now been shown to ameliorate pulmonary fibrosis, demonstrating the generalizability of this approach in organ fibrosis broadly (150, 151). Generating CAR T cells ex vivo is cumbersome, but Rurik and colleagues demonstrated that T cells can be transiently reprogrammed in vivo by injection of mRNA-containing lipid nanoparticles that target T cells with anti-CD5 on the nanoparticle surface (Figure 3) (152). In vivo T cell–targeted lipid nanoparticles reduced fibrosis and restored cardiac function in the pressure-overload model of cardiac injury. In vivo anti-FAP CAR T therapy also reduced liver fibrosis in MASH (153). Collectively, these studies provide the grounds for optimism that a new therapeutic era is dawning for the treatment of fibrotic diseases.

## Conflict of interest

BDH reports ownership of, stock options from, and income from Borealis Biosciences; stock options for Chinook Therapeutics, a Novartis Company; and research support from Mediar Therapeutics. BDH is listed as an inventor on five issued patents (61/458,140, 61/437,971, 61/973,554, 62/011,259, 62/170,083) and a pending patent (19/573,513).

## Funding support

BDH from the NIH National Institute of Diabetes and Digestive and Kidney Diseases (R01DK136663, R01DK103740, and U54DK137332).

## Version 1 07/08/2026

Electronic publication
